# Epidemiological, Serological, and Virological Features of Dengue in Nha Trang City, Vietnam

**DOI:** 10.4269/ajtmh.17-0630

**Published:** 2018-01-08

**Authors:** Duong Le Quyen, Nguyen Thanh Le, Cao Thi Van Anh, Nguyen Binh Nguyen, Dong Van Hoang, Jacqui L. Montgomery, Simon C. Kutcher, Nguyen Hoang Le, Nguyen Tran Hien, Duong Thi Hue Kien, Maia Rabaa, Scott L. O’Neill, Cameron P. Simmons, Dang Duc Anh, Katherine L. Anders

**Affiliations:** 1 National Institute of Hygiene and Epidemiology, Hanoi, Vietnam;; 2 Institute for Vector Borne Disease, Monash University, Clayton, Australia;; 3 Wellcome Trust Major Overseas Programme, Oxford University Clinical Research Unit, Hospital for Tropical Diseases, Ho Chi Minh City, Vietnam;; 4 Department of Microbiology and Immunology, University of Melbourne, Doherty Institute, Melbourne, Australia

## Abstract

Vietnam is endemic for dengue. We conducted a series of retrospective and prospective studies to characterize the epidemiology of dengue and population mobility patterns in Nha Trang city, Vietnam, with a view to rational design of trials of community-level interventions. A 10-year time series of dengue case notifications showed pronounced interannual variability, as well as spatial heterogeneity in ward-level dengue incidence (median annual coefficient of variation *k* = 0.47). Of 451 children aged 1–10 years enrolled in a cross-sectional serosurvey, almost one-third had evidence of a past dengue virus (DENV) infection, with older children more likely to have a multitypic response indicative of past exposure to ≥ 1 serotype. All four DENV serotypes were detected in hospitalized patients during 8 months of sampling in 2015. Mobility data collected from 1,000 children and young adults via prospective travel diaries showed that, although all ages spent approximately half of their daytime hours (5:00 am–9:00 pm) at home, younger age groups (≤ 14 years) spent a significantly greater proportion of their time within 500 m of home than older respondents. Together these findings inform the rational design of future trials of dengue preventive interventions in this setting by identifying 1) children < 7 years as an optimal target group for a flavivirus-naive serological cohort, 2) children and young adults as the predominant patient population for a study with a clinical end point of symptomatic dengue, and 3) substantial spatial and temporal variations in DENV transmission, with a consequent requirement for a trial to be large enough and of long enough duration to overcome this heterogeneity.

## INTRODUCTION

Dengue is a vector-borne tropical infectious disease that poses a public health challenge in over 100 countries.^1^ The disease is caused by dengue viruses (DENVs), of which there are four serotypes: DENV1–4.^2,3^ Dengue is an acute systemic febrile illness in which a small proportion of cases develop life-threatening complications.^2–5^ An estimated 50–100 million people worldwide suffer from a clinically apparent DENV infection each year,^1,6^ of an estimated 390 million (95% credible interval [CI]: 284–528 million) total annual DENV infections.^1^ DENVs are transmitted between humans by *Aedes* mosquitoes, with *Aedes aegypti* the primary vector and *Aedes albopictus* a secondary vector. The dramatic increase in global dengue incidence over the past half century has been linked to rapid urbanization, increased geographic distribution of vector species, and increasing human movement within and between areas.^2,3^

Dengue is endemic in Vietnam, and incidence is higher and more consistent in the south than the north of the country. Interannual peaks and troughs of dengue incidence characterize the historical time series.^7,8^ Dengue is a notifiable disease, and disease control is coordinated by the Vietnam National Dengue Control Program. The national strategy is based on reduction in mosquito breeding sites, insecticide spraying, management, and treatment of symptomatic cases.^9^ Over the last two decades, dengue case fatality rates have declined and are now very low.^10,11^ However, as in many other countries, there is little evidence that Vietnam has reduced the burden of morbidity from dengue, let alone eliminated dengue as a public health problem. A vaccine for dengue, Dengvaxia^®^, developed by Sanofi Pasteur, was trialled in Vietnam but is yet to be licensed locally and has a particularly challenging use profile.^12–15^

Other arboviruses are emerging in Vietnam. For example, four chikungunya and two Zika cases were detected in southern Vietnam in 2012 and 2013 respectively,^16^ and there have been repeated reports of Zika in international travellers to Vietnam.^17,18^ However, the scale and geographic extent of Zika and chikungunya virus transmission in Vietnam are poorly understood in part because diagnostic tests for these pathogens are not widely available.

Although vector control remains central to the control of arbovirus transmission, there is little field evidence to enable prioritization of different operational methods.^19^ Randomized trials provide the most unbiased estimates of intervention effect, and two alternative designs proposed for trials of vector control interventions are a classical parallel two-armed cluster randomized trial or a stepped-wedge cluster randomized trial.^20^ The spatial and temporal heterogeneity commonly observed in dengue incidence presents a challenge to both designs. Untreated ‘control’ areas should be close enough and similar enough to intervention areas to experience comparable dengue risk, but far and large enough to avoid contamination between treated and untreated areas. Temporal variation in disease incidence introduces uncertainty in the length of a trial required to capture sufficient illness events. Given the day-feeding behavior of *Ae. aegypti*,^3^ the daytime mobility of the resident population will also determine the proportion of the time spent outside the individuals’ assigned ‘intervention’ or ‘control’ zone and will therefore influence the power of the study to measure the effect of a cluster-randomized intervention.^21^ A sound understanding of baseline dengue epidemiology and human mobility patterns in prospective sites for field trials of dengue preventive interventions is therefore an essential prerequisite for rational trial design.^20^

Here, we retrospectively describe the epidemiological history of dengue in Nha Trang city and prospectively report the virological and serological profiles of dengue patients and healthy children, respectively. In addition, we describe the mobility profiles of children, adolescents, and young adults. These data will provide a baseline and critical information to guide the design of intervention trials to reduce arbovirus transmission in this setting.

## MATERIALS AND METHODS

### Study area.

**Figure 1.**
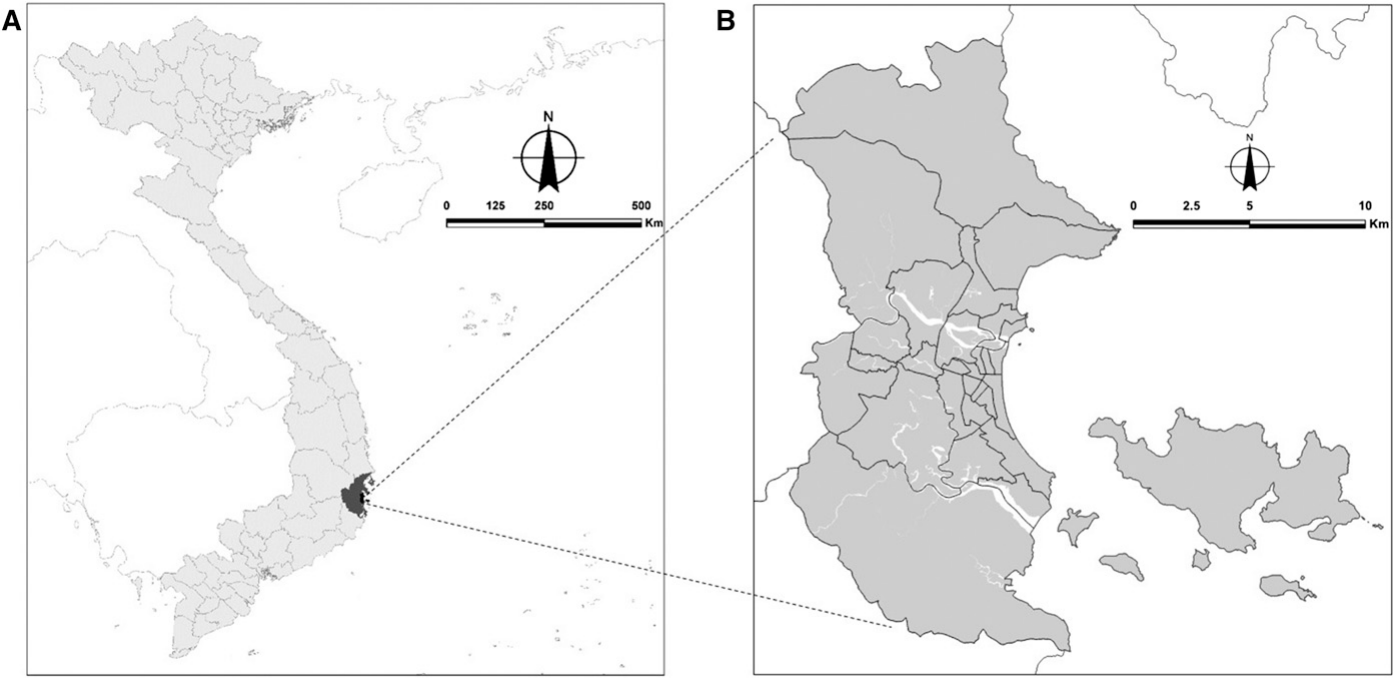
Map of Vietnam with Khanh Hoa province in dark gray (**A**) and map of Nha Trang city showing administrative boundaries of its 27 wards (**B**).

Nha Trang city is a coastal town in the southern central region of Vietnam (Figure 1A). In 2015, the population of the city was ∼415,000.^22^ The city covers an area of 252.6 km^2^ and is divided into 27 wards for administrative management (Figure 1B).

### Population data.

Demographic data including the total population and the population by ward from 2006 to 2015 were obtained from the General Statistics Office of Nha Trang city.^22^ Population data for 2016 have not yet been published, so were estimated by applying the average rate of population change over 2011–2015 to the 2015 data. Annual population data by age were not available, so were estimated by applying the age distribution from the 2009 National Census^23^ to annual population totals.

### Dengue case surveillance.

Dengue cases were notified by the community health stations and public hospitals to the Nha Trang Health Center on a daily basis, in accordance with the requirements and guidelines of the Vietnam National Dengue Control Program. Dengue case notifications are based on clinical signs and symptoms, according to the following criteria: sudden onset of fever lasting for 2–7 days and at least two of the following signs or symptoms: 1) hemorrhagic manifestations; 2) headache, loss of appetite, nausea, and vomiting; 3) rash or flushed skin; 4) pain in muscle, joint, or behind the eyes; 5) fatigue or restlessness; and/or 6) abdominal pain.^9^ The reported cases include both outpatients and inpatients.

### Dengue serology.

A cross-sectional population serosurvey was conducted in August and September 2015. Blood samples were collected from 451 children aged 1–10 years, living in 27 wards of Nha Trang, who were randomly selected using two-stage cluster sampling.^24^ The first sampling unit was hamlet, in which 52 hamlets were randomly selected (from a total of 360 hamlets in Nha Trang) with a probability proportional to the size.^25^ Within each hamlet, a list of all residents aged 1–10 years was obtained; 8–10 children were randomly selected from the list, and their guardians were invited to provide written informed consent for participation in the serosurvey. A 2 mL of venous blood sample was collected into an EDTA tube and transported daily to the laboratory at the Pasteur Institute of Nha Trang. Plasma was separated by centrifugation and stored at −20°C until testing by Panbio Dengue Immunoglobulin G (IgG) Indirect enzyme-linked immunosorbent assay (ELISA) (Alere, Australia), according to the manufacturer’s instructions, to determine previous exposure to DENV. A random subset of 80 ELISA-positive and 20 ELISA-negative samples were then tested also by plaque reduction neutralization test (PRNT) for four serotypes of DENV and Japanese encephalitis virus (JEV)^26,27^ at the Armed Forces Research Institute of Medical Sciences in Thailand. JEV was included because of its antibody cross-reactivity with DENV. At the time of the study, the local health system had been implementing a free JEV vaccination program for children aged 1–5 years for about 1 year. Before that, children older than 1 year were able to access JEV vaccination as a paid service.

### Viral diversity.

A cross-sectional study was conducted in May–December 2015 in two hospitals in Nha Trang city, Khanh Hoa General Hospital (Infectious Disease ward; May–September 2015) and Khanh Hoa Tropical Disease Hospital (October–December 2015). Blood samples (2.5 mL) were collected from 200 clinically suspected dengue patients who showed positive NS1 rapid test results (SD Bioline Dengue NS1 Ag). Samples were tested by real-time quantitative reverse transcriptase polymerase chain reaction,^28^ and DENV serotype was identified in 190 samples. Among these 190 samples, 50 samples representative of serotypes, temporal distribution, and spatial distributions were selected for genome sequencing.

### Mobility.

A cross-sectional population-based survey was performed in September and October 2015 to determine the regular mobility patterns of children and young adults in Nha Trang city. The study period was during the school term, at a time of average temperature and rainfall, so we expected people’s daily activities during this period to be reasonably representative of their typical routine. One thousand residents of Nha Trang city aged 1–29 years were randomly selected by two-stage cluster sampling,^24^ using hamlets (*N* = 50) as the primary sampling units and households (*N* = 20 per hamlet) as the secondary units. Hamlets were sampled with a probability proportional to the size.^25^ Criteria for inclusion were age 1–29 years, Vietnamese nationality, residence in Nha Trang city for the previous year, residence at the current address for the following 2 weeks, and written consent to participate. Only one participant was enrolled per household. If a sampled household had more than one eligible person, the Kish selection method was used to select one household member.^29^

Because the planned analyses of mobility data were descriptive and exploratory in nature, and we had no baseline data from which to estimate the distribution of the respondents’ mobility, we did not attempt a formal sample size calculation for the mobility survey. Instead, we pragmatically applied the same target sample size as for the seroprevalence survey (*N* = 500), for each age group (children 1–14 years and young adults 15–29 years).

Participants were interviewed at enrollment to record demographic information and routine activities and asked to complete a structured written travel diary for seven consecutive days from the day of enrollment, which captured details of the places visited between 5:00 am–9:00 pm each day, including duration of the visit. Study staff either visited participants at home or contacted them by phone, every second day, to prompt them to complete the travel diary. At the end of 7 days, study staff returned to review and collect the travel diary and record the location of each unique place visited into Google Earth, to derive geocoordinates. Travel diary data were later entered into a web-based database (iFormBuilder; Zerion Software, Herndon, VA) and linked to the geocoordinates. Google Earth was used to measure the distance from home to each location. Travel diary data were aggregated to calculate the total duration of time spent at each unique location over a week, for each participant. The proportion of daytime hours spent at home and at increasing distances from home was calculated by aggregating the hours spent at all locations within each distance interval (100 m bins between 0 and 2 km from home) and dividing by the total hours documented in the travel diary, for each respondent. Summary statistics were then produced within each age group (1–5 years; 6–10 years; 11–14 years; 15–17 years; 18+ years, based on school age groupings), and the Kruskal–Wallis rank-based nonparametric test was used to compare the distribution of the individual participants’ proportion of time spent at home, and within 500 m or 1 km of home, between age groups.

### Human research ethics approvals.

All the studies received ethical clearance from the Institutional Review Board at Vietnam National Institution of Hygiene and Epidemiology (NIHE). The research activities are part of a long-term collaboration between NIHE and the Eliminate Dengue Program, Monash University, Australia.

## RESULTS

### Trends in dengue case notifications.

**Figure 2.**
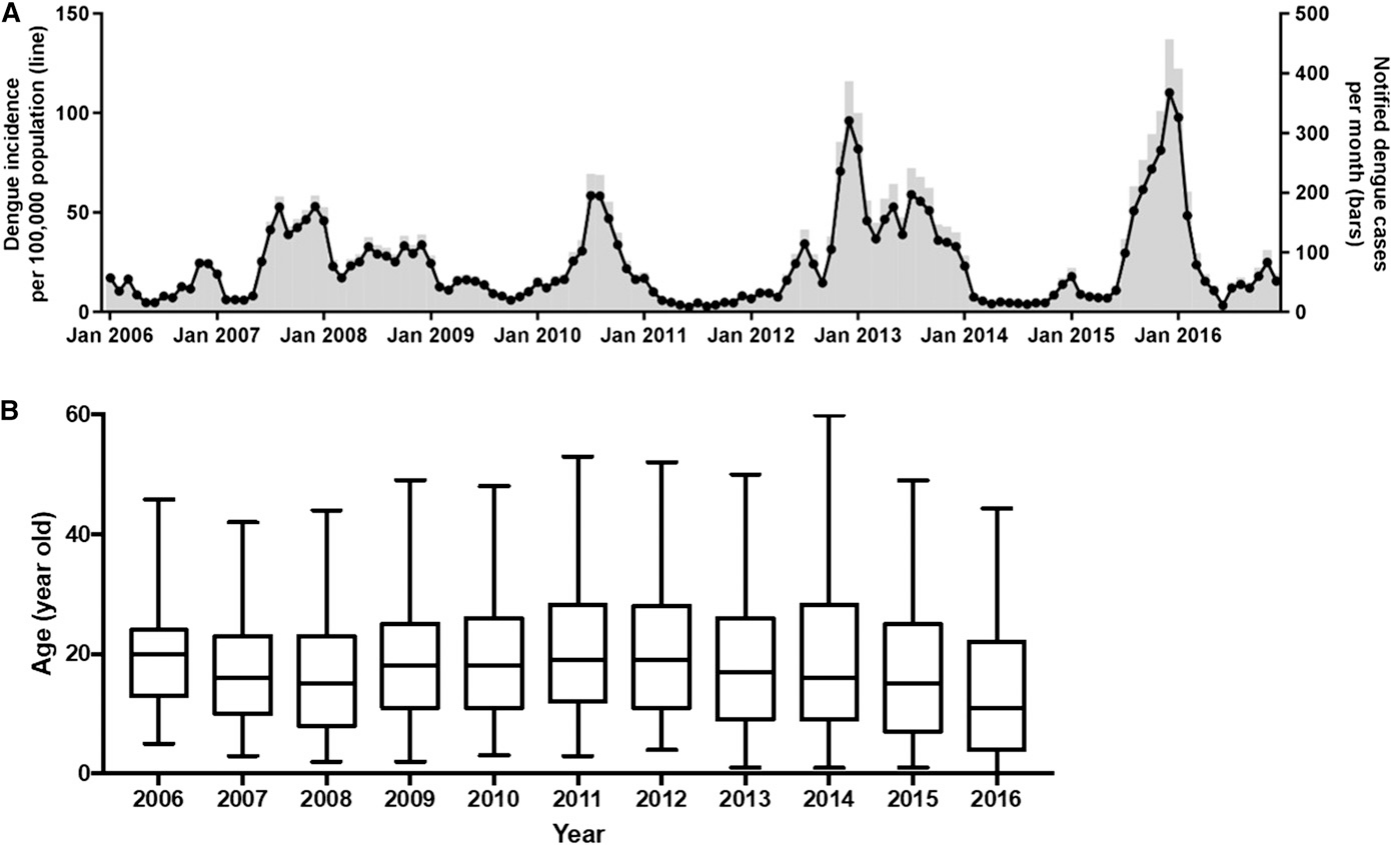
Dengue cases in Nha Trang notified to Nha Trang Health Center from 2006 to 2016 (11 years). The case definition for reporting purposes is described in the Materials and Methods. (**A**) Notified dengue incidence (black line) and case counts (shaded bars) by month from 2006 to 2016. (**B**) The age distribution of notified dengue cases each year. There are some years with a high percentage of missing data on the age of notified cases: 2009 (24.8%), 2007 (15.8%), and 2006 (11.4%).

From 2006 to 2016, a total of 12,655 dengue cases were notified in Nha Trang city, ranging from 285 to 2,335 cases per year. Peaks in annual notified dengue case numbers occurred in 2013 (2,335 cases) and 2015 (1,928 cases). The annual incidence ranged from 72 to 572 cases per 100,000 population (median 345 cases/100,000). The coefficient of variation (CoV = standard deviation (SD)/mean) in incidence over the 11 years 2006–2016 was 0.55 on an annual basis and 0.34 for two-yearly blocks (which may better reflect the follow-up period in an intervention trial) between 2006 and 2015. Figure 2 shows the number and per capita incidence of notified dengue cases by month over the period of 11 years. Cases were reported year-round, but with pronounced epidemic peaks occurring most commonly in July–August or November–January. A multiannual cycle of one or two high-incidence years followed by a pronounced trough was observed. An overall increase in the magnitude of dengue outbreaks over time was evident.

The median age of notified dengue cases ranged from 11 to 20 each year (Figure 2B). Although dengue cases were also reported among older adults, most dengue cases were in children and young adults, with the highest age-specific incidence in those aged 5–24 years (Supplemental Figure 1). In the most recent 4 years, the peak incidence seems to have shifted toward younger age groups, with children younger than 5 years experiencing comparable incidence rates as older children (Supplemental Figure 1). Although only 22.6% of the Nha Trang city population is 14 years of age or younger,^23^ this age group accounts for 44.1% of notified dengue cases over the 11-year period (range: 29.0–60.7%).

### Spatial patterns in dengue incidence.

**Figure 3.**
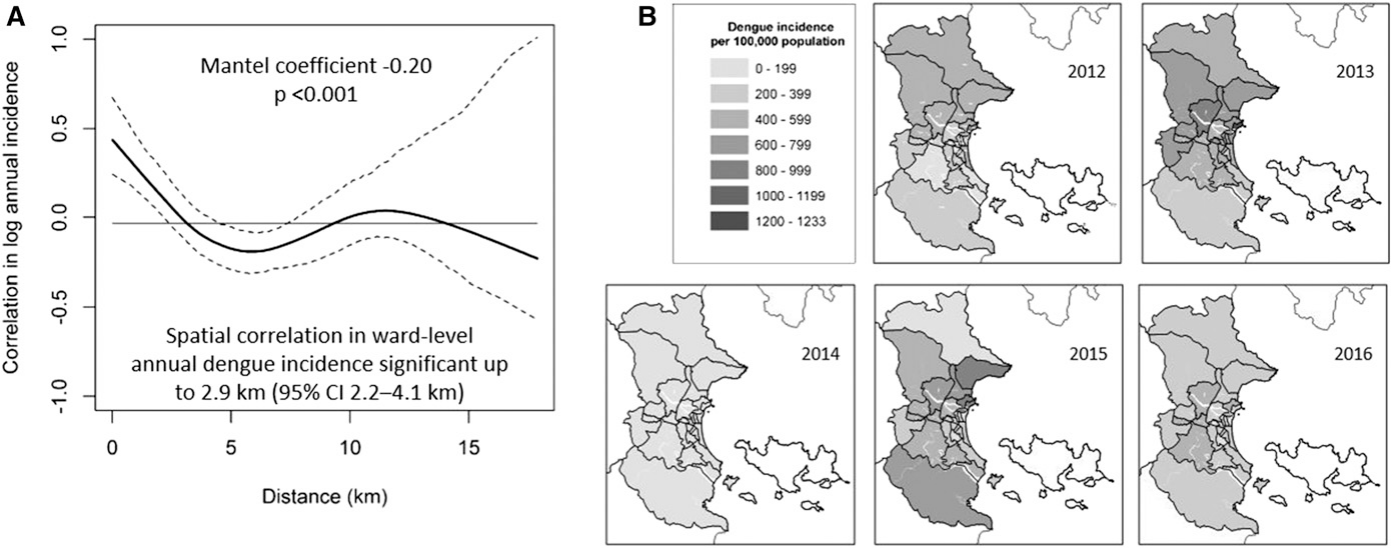
(**A**) Spatial correlation in dengue incidence in Nha Trang city, 2006–2016. The pairwise correlation (solid line) in log-transformed and standardized annual dengue incidence between 27 wards in Nha Trang city, as a function of the distance between the centroids of those wards. Dashed lines represent 95% confidence intervals, and the horizontal line is the overall correlation in ward-level annual dengue incidence across Nha Trang city. (**B**) Maps of Nha Trang city showing the annual notified dengue incidence (per 100,000 population) in each ward for the last 5 years (2012–2016).

Spatial correlation was observed in ward-level annual dengue incidence over the 11-year period (Figure 3A, *P* < 0.001, Mantel test). Dengue incidence was significantly more correlated between wards at a distance of 2.9 km or less (between ward centroids), compared with wards at a greater distance, and correlation increased with increasing proximity. Dengue cases were reported year-round in all 27 wards of Nha Trang city. In peak dengue years (e.g., 2012, 2013, and 2015), the highest dengue incidence was seen in the central urban wards (Figure 3B); however, no consistent patterns were observed across the time series in terms of which wards were most affected.

Overall spatial heterogeneity in dengue incidence was quantified by the CoV (CoV = SD/mean) in ward-level notified dengue incidence each year, 2006–2016, and also in two-yearly intervals, which may better reflect the follow-up period in a prospective study. The CoV ranged from 0.34 to 0.86 (median 0.47) annually and 0.31–0.54 (median 0.40) for two-yearly windows, when all 27 wards of Nha Trang were included. Restricting to only the 19 contiguous urban wards resulted in reduced spatial variability: CoV 0.21–0.75 (median 0.39) for annual windows and 0.21–0.49 (median 0.38) for two-yearly windows.

#### Cross-sectional serosurvey in children aged 1 to 10 years.

**Figure 4.**
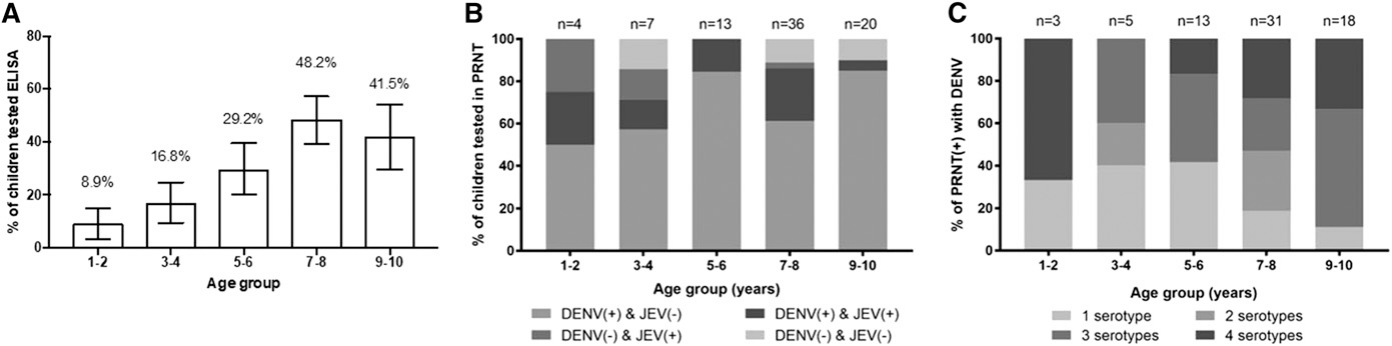
Age-stratified dengue seroprevalence in children aged 1–10 years in Nha Trang city. Venous blood samples were collected via a cross-sectional survey of 451 children residing in Nha Trang city in August/September 2015. (**A**) Bars show the proportion of samples positive by Panbio Dengue IgG Indirect enzyme-linked immunosorbent assay (ELISA) by 2-year age group, with 95% binomial confidence intervals. (**B**) Age-stratified proportion of children possessing virus neutralizing antibodies (PRNT50 titer ≥ 40) to dengue virus (DENV) or JEV. Plaque reduction neutralization test results are from a randomly selected subsample of 80 children who tested positive in the Panbio Dengue immunoglobulin G (IgG) Indirect ELISA shown in (**A**). Shown are the proportion of children to test positive against DENV-1–4 (any serotype) alone, JEV alone, DENV-1–4 and JEV, or neither virus. One sample that was negative in the Panbio IgG Indirect ELISA, but positive in the DENV-1–4 PRNT, is not shown. (**C**) Homotypic and heterotypic virus neutralizing antibody profiles to DENV1–4. Shown are the proportion of children, among those with any virus neutralizing antibodies against DENV1–4 (*N* = 70), who possessed monotypic or multitypic antibody profiles. JEV = Japanese encephalitis virus; PRNT = plaque reduction neutralization test.

Among 451 plasma samples from children aged 1 to 10 years, 131 (29.0%) had serological evidence (i.e., tested positive in the Panbio Dengue IgG indirect ELISA) of a previous exposure to a flavivirus, either through infection or vaccination (e.g., JEV vaccination). There was a clear trend for increasing seropositivity with increasing age (Figure 4A). Of the randomly selected 80 ELISA test–positive samples that were tested in virus neutralization assays, 70 (87.5%) were positive to at least one DENV serotype, 17 (21.3%) were positive to JEV, and 14 (17.5%) were positive to both JEV and DENV. There were seven samples negative in the PRNT to both DENV and JEV. Among the 20 ELISA test–negative samples tested by PRNT, one was positive to DENV-1 (PRNT titer = 101) and one to JEV (PRNT titer = 489). These samples with presumed false negative ELISA results were not included in the following analyses. The age-stratified PRNT profile to DENV and JEV is shown in Figure 4B. Among those with PRNT antibodies to DENV, the proportion with a multitypic profile was, as expected, higher in older children compared with young children (Figure 4C): 42/50 (84%) 7–10-year-olds had multitypic neutralizing antibody versus 12/20 (60%) 1–6-year-olds (prevalence ratio 1.4 [95% CI 0.96–2.0], Fisher’s exact *P* = 0.056). Neutralizing antibodies to DENV-1 (80% or 56 samples) were most prevalent, followed by DENV-2 and DENV-3 (78.6% or 55 samples each serotype), and then DENV-4 (30.0% or 21 samples). Collectively, these data suggest that DENV infection likely accounts for much of the flavivirus exposure history in Nha Trang city.

#### A contemporary snapshot of DENV genetic diversity in Nha Trang.

We identified 190 viremic dengue cases among 200 enrolled acute dengue patients (all NS1 rapid test positive) enrolled in 2015. All four serotypes were detected; DENV-1 (*N* = 105), DENV-2 (*N* = 67), DENV-3 (*N* = 16), and DENV-4 (*N* = 3). From these 190 viremic samples, a subset of 49 cases were selected for genome length sequencing and this yielded full or partial genome sequences for DENV-1 (*N* = 29), DENV-2 (*N* = 15), and DENV-4 (*N* = 1). Maximum likelihood trees derived from envelope sequences indicated that DENV-1 belonged to the Genotype I lineage; DENV-2 were of the Asian I lineage; and DENV-4 were of the Genotype I lineage (Supplemental Figure 2). These Nha Trang sequences formed their own clade, suggestive of sustained local transmission throughout the season, and were distinct from their nearest neighbors, which were sequences from southern Vietnam.

#### Mobility of children, adolescents, and young adults in Nha Trang.

**Figure 5.**
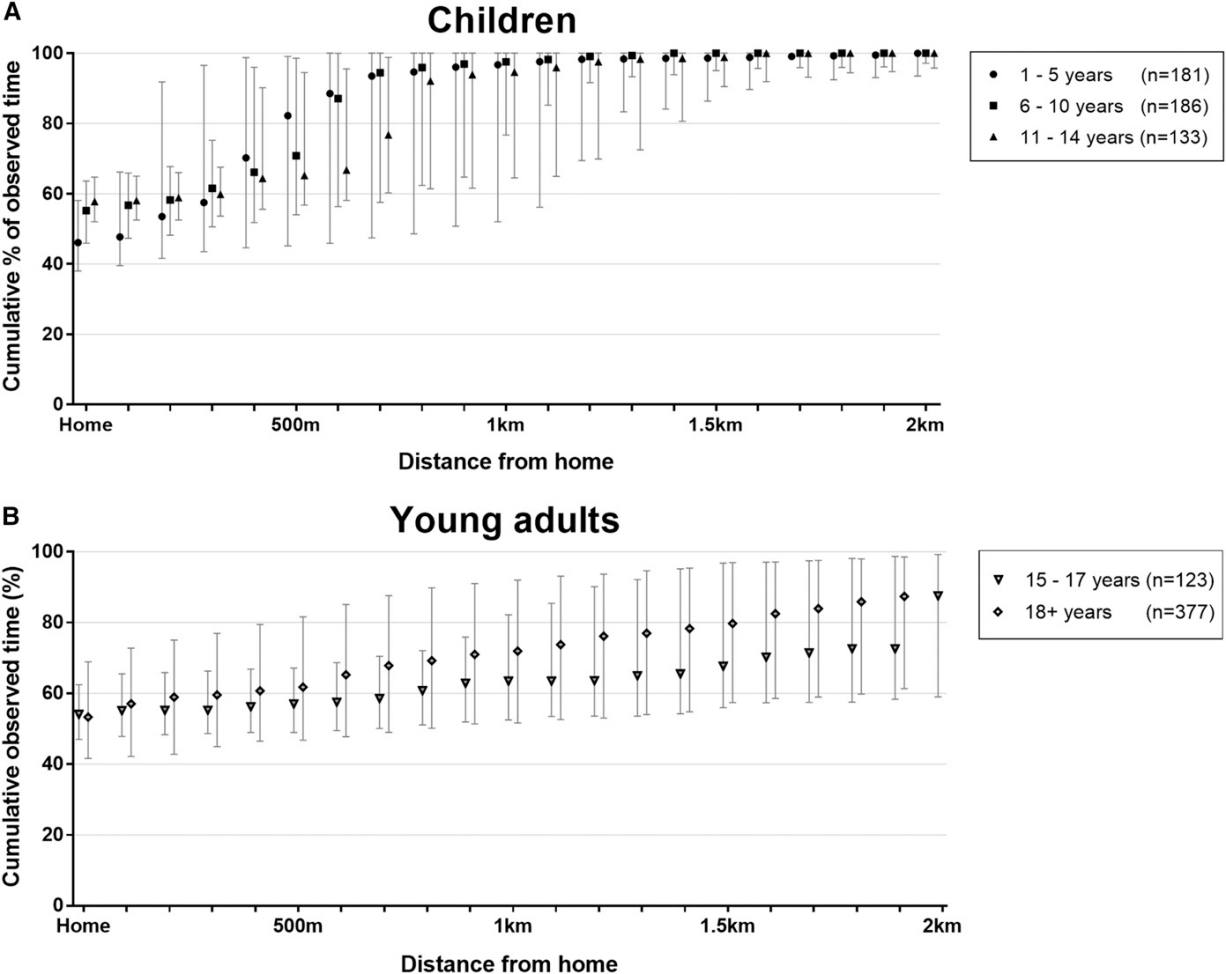
Mobility of Nha Trang residents aged 1–29 years, determined by self-reported travel diaries. Graphs show the median and interquartile range of the proportion of time (5:00 am to 9:00 pm aggregated over seven consecutive days) that 1,000 respondents spent at home and within increasing distances from home, by age group: (**A**) contains data for children younger than 5 years, 6–10 years, and 11–14 years, who would generally be expected to attend local kindergartens or schools inside the administrative area where they are resident. (**B**) contains data for respondents 15 years and older, who may study or work at various locations across the city.

Human mobility represents a challenge to cluster randomized trials of interventions against dengue. To gain insights into mobility, travel diaries were collected from 1,000 study participants for seven consecutive days, representing 112,000 hours from 5:00 am to 9:00 pm. Figure 5 shows the cumulative distribution of participants’ time spent at home and at increasing distances from home, as the median and interquartile range of total observed time per participant, by age group. Both child and adult respondents spent on average approximately half of their daytime hours at home. Younger age groups, however, spent a greater proportion of their time closer to home: the median proportion of daytime hours spent within 500 m of home was 82%, 71%, and 65% for children 1–5, 6–10, and 11–14 years, respectively, compared with 57% and 62% for young adults 15–17 years and ≥ 18 years, respectively. A Kruskal–Wallis rank test was performed to compare the proportion of time spent within 500 m from home by respondents in different age groups, which showed there was a statistically significant difference among the five groups: χ^2^ = 44.42, *P* = 0.0001. This age association was even more pronounced for movements within 1 km from home (median ≥ 95% of daytime hours for all child age groups versus 63% and 72% in 15–17 and ≥ 18 years respectively; Kruskal–Wallis χ^2^ = 122.72, *P* = 0.0001).

## DISCUSSION

Here, we have demonstrated three critical features of dengue in Nha Trang city relevant for planning field trials of interventions to reduce arbovirus transmission. First, there is pronounced temporal and spatial heterogeneity in dengue incidence across the city. Second, children and young adults are the populations most at risk of infection and disease. Third, all four DENV serotypes cocirculate in Nha Trang within a single season.

Seasonal peaks in dengue incidence occurred in most years between 2006 and 2016, coincident with the wet season. Consistent with reports from elsewhere in Vietnam^7,8^ and other endemic settings,^30–32^ there is substantial interannual variability in the magnitude of these seasonal peaks. In high-incidence years, upwards of 2,000 dengue cases were notified in Nha Trang, representing a substantial burden on the local health services. In comparing the incidence of notified dengue in Nha Trang (median annual incidence 345/100,000 population) to other reports from Vietnam, it is important to note that the Nha Trang surveillance data include ambulatory cases reported from outpatient settings as well as inpatients, whereas data reported previously from southern Vietnam (median annual incidence 232/100,000 population^8^) included only notifications of hospitalized cases. The median age of notified dengue cases in Nha Trang was 11–20 years over the study period, which is younger than previously reported in northern Vietnam^7^ and similar to estimates from hospitalized cases in southern Vietnam.^10,33^ Together, these data are consistent with an overall increase in intensity of DENV transmission from the north of the country to the south.

Serological results showed that approximately half of the children aged 7–8 years in Nha Trang have DENV-reactive antibodies indicative of a past flavivirus infection, with virus neutralization assays confirming that the majority of this seropositivity can be attributed to previous DENV infection. As expected, seropositivity increased with age, and older children were more likely to have multitypic neutralizing antibody responses, consistent with past exposure to more than one DENV serotype. This population immune profile has implications when considering implementation of the recently licensed Dengvaxia dengue vaccine in this population, as the vaccine is recommended for use only in target age groups with seroprevalence ≥ 70%.^15^ Our data indicate that children ≤ 10 years of age would therefore not be a suitable target population for vaccination in Nha Trang. These serological data are also relevant when considering the design of cluster randomized trials to evaluate vector control interventions such as the deployment of *Wolbachia*-infected mosquitoes.^34–36^ One potential approach to evaluating an efficacy end point in such trials is to measure seroconversion rates in prospective cohorts of children, serologically naive at baseline, who are resident in treated versus untreated areas.^20,37^ The serosurvey results reported here indicate that such a study would need to target recruitment to young children < 7 years in order for a large majority of the target population to be serologically naive. A further complication to this study design is the increasing uptake of JEV vaccination since the implementation from 2014 of a free JEV vaccination program for children aged 1–5 years. JEV vaccination induces antibodies that are cross-reactive in the DENV IgG indirect ELISA and distinguishable only by a more costly and lower throughput neutralization assay.

We have previously used data from Thailand to inform simulations that measured the relative contributions of spatial, temporal, and residual variations (i.e., variation that cannot be explained by systematic spatial or temporal variation) on cluster number requirements in parallel and step-wedge intervention trial designs for dengue.^20^ Unsurprisingly, the larger the variance in dengue incidence that exists in time and space between “clusters” (a priori defined units of geographic area, typically half of which will be randomly allocated to the intervention), then the greater the number of clusters (and potentially also participants per cluster) that are required to overcome the nonindependence of study participants within each cluster.^38^ In Nha Trang, the spatial variation (median CoV = 0.47) and temporal variations (CoV = 0.55) in dengue incidence were very similar to those described for the case study of dengue in Thailand (0.52 and 0.53, respectively),^20^ although it should be noted that different (model-based) methods were used in the previous work, and therefore these values are not directly comparable. In short, design of any future cluster randomized trial in Nha Trang needs to account for high baseline levels of intercluster variability when estimating the minimum sample size (numbers of clusters and participants).

Routine daily movements of the human population under study present a challenge to cluster-randomized trials of an area-based intervention like *Wolbachia*, especially where clusters are contiguous. If clusters are too small and/or movement too great, this will reduce the ability to detect a treatment effect, as all participants could—at the extreme—spend half of their exposure time in treated and half in untreated clusters. We found that the median proportion of daytime hours spent at home was between 46–58% for all age groups. As expected, high-school aged children 15–17 years and young adults spent more of their time further from home compared with younger age groups, a pattern that has also been observed in other settings.^39^ Inferring optimal minimum cluster size from these data is complicated by directionality, socially structured movement,^40^ and other nonrandom factors that shape real-world human movement patterns, but our findings indicate crudely that clusters should have a radius of at least 500 m, ideally greater, to capture most participants’ exposure time to day-biting *Ae. aegypti*. Restricting enrollment in a prospective pediatric cohort to only those children who reside and attend school within the same cluster or adopting a ‘fried-egg’ approach where only those resident in the center of each cluster are included in disease surveillance^41^ are potential ways to reduce the impact of human mobility on the observed treatment effect. If movement outside the cluster of residence during the period of likely exposure can be reliably recorded, this can also be adjusted for in the analysis. However, this relies on retrospective elicitation of travel histories with inherent limitations to recall.

In Nha Trang city, all four DENV serotypes were detected among hospitalized patients in an 8-month sampling period. Understanding the local distribution of circulating DENV serotypes is important for predicting the likely impact of interventions that are heterogeneous in their serotype-specific efficacy, as has been seen for both the Dengvaxia dengue vaccine^13,42^ and *Wolbachia*-mediated blocking of DENV infection in *Ae. aegypti*.^43^

We have demonstrated in this series of descriptive studies that Nha Trang city in central Vietnam experiences a substantial, although annually variable, burden of disease due to dengue. The recent detection of Zika virus in *Ae. aegypti* mosquitoes in Nha Trang as well as in one symptomatic patient with no travel history^44^ increases the imperative for improved methods for *Aedes* control in this and other dengue endemic settings. A robust trial design for existing and novel strategies for arbovirus vector control is complicated by the spatial and temporal heterogeneity inherent to dengue epidemiology, the young age of affected populations that would be recruited into a trial, and the regular movement of study populations across cluster boundaries.^21^ We have characterized here these features of dengue epidemiology and human mobility in Nha Trang city, as a first step toward the rational design of future trials of dengue preventive interventions in this setting, including potential field trials to measure the impact of *Wolbachia*-infected mosquitoes on dengue and other arboviruses.

## Supplementary Material

Supplemental Figures.
