# Supplementary material for: Epidemiological, Serological, and Virological Features of Dengue in Nha Trang City, Vietnam
Source: Am J Trop Med Hyg. 2018 Jan 8;98(2):402–9. doi: 10.4269/ajtmh.17-0630 (PMC5929208; doi:10.4269/ajtmh.17-0630)
Supplement: Supplementary file 1 [file tpmd170630.SD1.pdf]

Age	2006	2007	2008	2009	2010	2011	2012	2013	2014	2015	2016
0-4	71	311	508	112	277	58	239	784	162	1,010	964
5-9	192	583	815	186	615	115	668	1,231	159	1,044	768
10-14	255	789	831	268	721	112	652	1,202	207	921	631
15-19	254	461	511	224	525	129	543	643	101	474	293
20-24	374	579	349	187	560	125	590	773	130	638	214
25-29	116	259	321	102	382	63	385	494	69	467	206
>30	43	76	103	41	124	33	159	225	42	178	95

Supplemental Figure 1. Heat map showing the age-specific incidence (per 100,000 population) of notified dengue cases in Nha Trang city each year, 2006–2016. Note that several years have missing age information for a substantial proportion of cases, which therefore are not included in these data: 2009 (24.8%), 2007 (15.8%), and 2006 (11.4%).

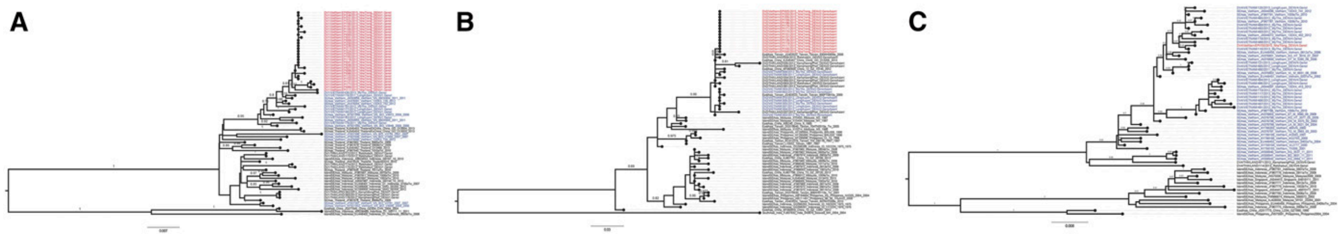

Supplemental Figure 2. Midpointrooted maximum likelihood phylogeny of E gene sequences of (**A**) DENV-1, (**B**) DENV-2, and (**C**) DENV-4 from Nha Trang city, and a representative sample of Southeast Asian DENV sequences. Phylogenetic trees were constructed in PHYLIP (GTR + $\Gamma(4)$ + I model, 1,000 bootstraps). The tip labels for the Nha Trang city sequences generated in this study are shaded red, whereas sequences from DENV sampled in southern Vietnam are shaded blue. Bootstrap values are shown for nodes with bootstrap support ≥ 60%. Scale bars represent the number of nucleotide substitutions per site. DENV = dengue virus.
